# Associations of *HLA* Polymorphisms with Anti-SARS-CoV-2 Spike and Neutralizing Antibody Titers in Japanese Rheumatoid Arthritis Patients Vaccinated with BNT162b2

**DOI:** 10.3390/vaccines11020404

**Published:** 2023-02-09

**Authors:** Takashi Higuchi, Shomi Oka, Hiroshi Furukawa, Shigeto Tohma

**Affiliations:** 1 Department of Rheumatology, National Hospital Organization Tokyo National Hospital, 3-1-1 Takeoka, Kiyose 204-8585, Japan; 2 Department of Nephrology, Ushiku Aiwa General Hospital, 896 Shishiko-cho, Ushiku 300-1296, Japan

**Keywords:** *HLA*, anti-SARS-CoV-2 spike antibody, anti-SARS-CoV-2 neutralizing antibody, rheumatoid arthritis, vaccination

## Abstract

Severe acute respiratory syndrome coronavirus 2 (SARS-CoV-2) causes Coronavirus Disease 2019. Anti-SARS-CoV-2 spike (S) and neutralizing antibodies (Abs) are measured to evaluate the efficacy of vaccines. Human leukocyte antigen (*HLA*) may be associated with vaccine efficacy. Here, we investigated the association of *HLA* polymorphisms with the production of anti-SARS-CoV-2 S or neutralizing Abs in vaccinated rheumatoid arthritis (RA) patients in Japan. Genotyping of *DRB1* and *DQB1* was conducted in 87 Japanese RA patients vaccinated with BNT162b2. Associations of allele or haplotype carrier frequencies with anti-SARS-CoV-2 S or neutralizing Abs were examined. DRB1*12:01 was significantly positively associated with the production of S Ab (*p* = 0.0225, odds ratio [OR] 6.08, 95% confidence interval [CI] 1.32–28.03). The DQB1*03:01 allele carrier frequency tended to be higher in high responders of S Ab. Allele carrier frequencies of DRB1*15:01 (*p* = 0.0102, OR 9.26, 95% CI 1.65–52.01) and DQB1*06:02 (*p* = 0.0373, OR 7.00, 95% CI 1.18–41.36) were higher in responders of neutralizing Ab. Haplotype and two-locus analyses of *DRB1* and *DQB1* suggested that *DRB1* alleles were the primary drivers of these associations. Logistic regression analysis showed associations of these alleles independent of clinical characteristics. Independent associations were found between *HLA* alleles and anti-SARS-CoV-2 Ab production by vaccinated RA patients.

## 1. Introduction

Coronavirus Disease 2019 (COVID-19) is caused by severe acute respiratory syndrome coronavirus 2 (SARS-CoV-2), which emerged as a pandemic starting in December 2019 [1]. A quantitative reverse transcription polymerase chain reaction is conducted for a definitive diagnosis. Serum anti-SARS-CoV-2 nucleocapsid (N) antibody (Ab) titers are quantified in order to detect a previous history of COVID-19. The presence of anti-SARS-CoV-2 spike (S) Ab in sera of vaccinated individuals is indicative of vaccine responsiveness when the S protein sequence of SARS-CoV-2 is used in COVID-19 mRNA vaccines. Anti-SARS-CoV-2 neutralizing Abs are titered to estimate the protective efficacy of Abs against infection. Blocking enzyme-linked immunosorbent assays has been employed recently to detect neutralizing Abs [2,3,4]. It is thought that the long-term production of neutralizing Abs is important for protection against SARS-CoV-2 infection. Humoral responses against COVID-19 mRNA vaccines are quite variable between individuals, influenced inter alia by age, sex, the time between the last vaccination and blood collection, or adverse reactions to the vaccine [5,6,7]. Levels of anti-SARS-CoV-2 S and neutralizing Abs were reported to be lower in patients with rheumatic diseases including rheumatoid arthritis (RA) relative to healthy controls [8,9,10].

Human leukocyte antigen (*HLA*) polymorphisms were reported to be associated with the efficacy of vaccines against measles [11,12], hepatitis B [13,14,15], or influenza [16,17]. However, few studies on the association of *HLA* with levels of anti-SARS-CoV-2 S Abs have been reported [18,19]. Hence, we investigated the association of *HLA* polymorphisms with levels of anti-SARS-CoV-2 S-specific or neutralizing Abs in vaccinated RA patients in Japan.

## 2. Materials and Methods

### 2.1. Patients and Sera

Eighty-seven RA patients were recruited at the National Hospital Organization Tokyo National Hospital. All RA patients fulfilled the American College of Rheumatology Criteria for RA [20] or Rheumatoid Arthritis Classification Criteria [21]. None of the RA patients had any history of COVID-19 before serum collection. All these patients had been vaccinated twice against SARS-CoV-2 with mRNA vaccine BNT162b2 (Pfizer, New York, NY, USA), and sera were then collected prior to the third vaccination.

The study protocol was reviewed and approved by The Research Ethics Committee of the National Hospital Organization Tokyo National Hospital. Written informed consent was obtained from each RA patient. This study was conducted in accordance with the principles expressed in the Declaration of Helsinki.

### 2.2. Detection of Anti-SARS-CoV-2 N, S, and Neutralizing Abs

The IgG fraction of anti-SARS-CoV-2 N Abs was measured by Anti-SARS-CoV-2 N IgG chemiluminescent enzyme immunoassays (Fujirebio, Hachioji, Japan). At a cut-off value of 1.0 U/mL, none of the RA patients were positive for the anti-SARS-CoV-2 N Abs. Anti-SARS-CoV-2 S Ab titers were quantified by Elecsys Anti-SARS-CoV-2 S (Roche Diagnostics, Mannheim, Germany) detecting Abs including IgG against the receptor-binding domain on the S1 subunit of SARS-CoV-2. The cut-off value was 0.8 U/mL. RA patients with high levels of anti-SARS-CoV-2 S Abs were classified as high responders (S high, top 25th percentile of anti-SARS-CoV-2 S Ab distribution) or low responders (S low, bottom 75th percentile of anti-SARS-CoV-2 S Ab distribution). Anti-SARS-CoV-2 neutralizing Abs (neu) were quantified using SARS-CoV-2 Neutralization Antibody Detection Kits (Medical & Biological Laboratories Co., Ltd., Tokyo, Japan). The degree of neutralization was calculated as follows: (1-optical density value of sample/optical density value of blank) × 100 (%). RA patients with high levels of anti-SARS-CoV-2 neutralizing Ab were classified as high responders (Neu high, top 25th percentile of anti-SARS-CoV-2 neutralizing Ab distribution) or low responders (Neu low, bottom 75th percentile of anti-SARS-CoV-2 neutralizing Ab distribution). The results of anti-SARS-CoV-2 N, S, and neutralizing Ab titers for the RA patients were previously reported [22].

### 2.3. Genotyping

*HLA* genotyping of *DRB1* and *DQB1* loci was performed by the polymerase chain reaction with reverse sequence-specific oligonucleotide probes (WAKFlow HLA typing kits, Wakunaga, Akitakata, Japan), using the Bio-Plex system (Bio-Rad, Hercules, CA, USA).

### 2.4. Statistical Analysis

Data were analyzed by Fisher’s exact test using 2 × 2 contingency tables or Mann-Whitney’s U test. Deviation from Hardy-Weinberg equilibrium was detected by Genepop (http://genepop.curtin.edu.au/ (accessed on 15 November 2022) [23]. Associations of *HLA* allele, carrier frequencies or haplotype carrier frequencies were tested by Fisher’s exact test using 2 × 2 contingency tables. To estimate the primary associations of alleles, two-locus analysis was employed with Fisher’s exact test using 2 × 2 contingency tables. Multiple logistic regression analysis under the additive model was employed to examine whether *HLA* alleles are independently associated with the production of anti-SARS-CoV-2 S Abs or neutralizing Abs in RA patients. Simple linear regression analysis was also employed to estimate the association of a rheumatoid factor with anti-SARS-CoV-2 neutralizing Abs in RA patients.

## 3. Results

### 3.1. Clinical Characteristics of RA Patients

Clinical manifestations of RA in the patient cohort are listed in Table 1. SARS-CoV-2 S Ab low responders were older than the high responders. The mean interval between the last vaccination and blood collection was longer in the low-responder group.

### 3.2. Association of HLA with Anti-SARS-CoV-2 Abs in RA Patients

Genotyping of *DRB1* and *DQB1* was performed to compare allele or haplotype carrier frequencies in the RA patients (Table 2, Table S1). Deviation from Hardy-Weinberg equilibrium was found in the RA patients (*DRB1*: *p* = 0.0144, *DQB1*: *p* = 0.5187). DRB1*12:01 was significantly associated with the production of S Ab (*p* = 0.0225, odds ratio [OR] 6.08, 95% confidence interval [CI] 1.32–28.03). There was also an association of DQB1*03:01 with the production of S Ab, but this did not achieve statistical significance (*p* = 0.0583, OR 2.78, 95% CI 1.00–7.69). However, DQB1*03:02 was associated with lower production of S Ab (*p* = 0.0089, OR 0.07, 95% CI 0.00–1.16). The haplotype carrier frequency of DRB1*12:01-DQB1*03:01 (*p* = 0.0225, OR 6.08, 95% CI 1.32–28.03) was higher in the S Ab high responders, but because other haplotypes including DQB1*03:01 were not significantly associated with the production of S Ab, this suggests a primary role of DRB1*12:01.

The allele carrier frequency of DRB1*15:01 was higher in the highly neutralizing Ab responders (*p* = 0.0102, OR 9.26, 95% CI 1.65–52.01, Table 3, Table S2). The allele carrier frequency of DQB1*06:02 was also higher in the high responders (*p* = 0.0373, OR 7.00, 95% CI 1.18–41.36). The haplotype carrier frequency of DRB1*15:01-DQB1*06:02 (*p* = 0.0337, OR 7.00, 95% CI 1.18–41.36) was higher in the high responders of neutralizing Ab. A tendency towards an association of DRB1*15:01-DQB1*03:01 with the production of neutralizing Ab did not reach statistical significance (*p* = 0.2529, OR 9.14, 95% CI 0.36–232.79), suggesting the primary role of DRB1*15:01.

Because *DRB1* and *DQB1* are in strong linkage disequilibrium, a two-locus analysis was performed to determine which locus was primarily responsible for the associations (Table 4). The OR for DQB1*03:01 in S Ab high responders without DRB1*12:01 was 1.74 (*p* = 0.5052). The OR for DRB1*12:01 in high responders with DQB1*03:01 was 4.00 (*p* = 0.1936), suggesting that the primary association was with DRB1*12:01.

The two-locus analysis was also performed to elucidate the primary role of DRB1*15:01 and DQB1*06:02 in the production of neutralizing Ab (Table 5). The OR for DQB1*06:02 in the neutralizing Ab high responders with DRB1*15:01 was 0.60 (*p* = 1.0000). The OR for DRB1*15:01 in the high responders without DQB1*06:02 was 10.89 (*p* = 0.2222), again suggesting a DRB1*15:01 primary association.

### 3.3. Logistic Regression Analysis of HLA Alleles and Clinical Characteristics

To exclude the possibility of an influence of the clinical characteristics on the production of anti-SARS-CoV-2 Abs, conditional logistic regression analysis of HLA alleles and these characteristics was conducted (Table 6). The results of the logistic regression analysis of DRB1*12:01 and clinical characteristics suggested an independent association of DRB1*12:01 with the production of S Ab. Age remained associated with anti-SARS-CoV-2 S Ab levels when conditioned on the other factors.

The results of the logistic regression analysis of DRB1*15:01 and clinical characteristics also suggested an independent association of DRB1*15:01 with the production of neutralizing Ab (Table 7). The time between the last vaccination and blood collection remained associated with anti-SARS-CoV-2 neutralizing Ab levels when conditioned on the other factors. Thus, anti-SARS-CoV-2 S Ab titers were higher in RA patients with DRB1*12:01, while anti-SARS-CoV-2 neutralizing Ab levels were higher in those with DRB1*15:01.

Rheumatoid factor was weakly and negatively associated with the titer of neutralizing antibodies in the previous report (partial regression coefficient [PRC] −0.0003, 95% CI −0.0006~0.0000, *p* = 0.0390) [22]. The association between the rheumatoid factor and DRB1*15:01 was investigated via linear regression analysis. No significant association was detected (PRC −117.45, 95% CI −399.03~164.12, *p* = 0.4092).

## 4. Discussion

The present study revealed that DRB1*12:01 and DRB1*15:01 were associated with anti-SARS-CoV-2 S and neutralizing Ab levels, respectively, in Japanese RA patients vaccinated with BNT162b2. Although *DRB1* and *DQB1* are in strong linkage disequilibrium, haplotype and two-locus analyses of *DRB1* and *DQB1* suggested the primary roles of *DRB1* alleles. These associations were independent of the clinical manifestations of the RA patients.

The association of *HLA* with anti-SARS-CoV-2 S Abs had been previously investigated in 56 healthcare workers [19]; no association was detected. However, in silico DRB1*15:01 was predicted to have a higher number of strong binding peptides derived from the entire sequence of the SARS-CoV-2 S protein [19]. *HLA* associations with anti-SARS-CoV-2 S Abs had also been analyzed in 100 Japanese hospital workers [18] and *DQA1:03:03* was reported to be associated with the production of greater amounts of anti-SARS-CoV-2 S Abs. In that study, the association of DRB1*12:01 with higher levels of anti-SARS-CoV-2 S Abs was not reported. An additional consideration is that there is no linkage disequilibrium between DRB1*12:01 and *DQA1:03:03* in Japanese populations [24]. These results are therefore not consistent with those of the present study, but RA patients clearly constitute an immunologically different population. This could be the reason for such different results, because anti-SARS-CoV-2 S Ab is sometimes not produced at all in certain patients with rheumatic diseases after vaccination [25]. Although haplotype and two-locus analyses suggested the primary role of *DRB1* alleles, other genes in linkage disequilibrium with *DRB1*-*DQB1* loci might also increase the production of anti-SARS-CoV-2 Abs. Adult-onset Still’s disease was associated with DRB1*12:01 and DRB1*15:01 in Japanese populations [26,27]; several patients with adult-onset Still’s disease after being vaccinated against SARS-CoV-2 were reported [28,29,30]. It was known that drug-induced adverse effects are common in adult-onset Still’s disease. These data suggested that these *DRB1* alleles might confer higher drug responsiveness in conditions with inflammation.

The present study on the association of *HLA* with anti-SARS-CoV-2 S and neutralizing Ab in vaccinated RA patients does have some limitations. The sample size is modest and this is a single-center study performed in Japan. The distribution patterns of *HLA* alleles are different in other ethnic populations. Other loci in the *HLA* region would influence the production of anti-SARS-CoV-2 Abs. Larger multiethnic studies on the whole *HLA* region are warranted to confirm the findings of the present study. In the future, associations of *HLA* with the roles of memory B or memory T cells could be examined in vaccinated RA patients [10,31,32].

## 5. Conclusions

In conclusion, in the present study, independent associations of *HLA* alleles with anti-SARS-CoV-2 Ab levels were found in Japanese RA patients vaccinated with BNT162b2.

## Figures and Tables

**Table 1:** Characteristics of the RA study patients.

	S High	S Low	*p*	Neu High	Neu Low	*p*
Number	22	65		22	65	
Age, years (SD)	67.7 (10.6)	73.8 (8.1)	0.0147	73.0 (7.5)	72.0 (9.6)	0.7359
Male, *n* (%)	4 (18.2)	17 (26.2)	0.5707 *	7 (31.8)	14 (21.5)	* 0.3904
Corticosteroid administration, *n* (%)	8 (36.4)	25 (38.5)	1.0000 *	7 (31.8)	26 (40.0)	* 0.6138
csDMARDs administration, *n* (%)	17 (77.3)	39 (60.0)	0.1992 *	15 (68.2)	41 (63.1)	* 0.7986
bDMARDs administration, *n* (%)	1 (4.5)	10 (15.4)	0.2769 *	1 (4.5)	10 (15.4)	* 0.2769
tsDMARDs administration, *n* (%)	5 (22.7)	17 (26.2)	1.0000 *	4 (18.2)	18 (27.7)	* 0.5710
Interval between last vaccination and blood collection, days (SD)	145.6 (41.8)	143.5 (28.1)	0.6115	129.2 (27.5)	149.0 (31.8)	0.0370
Anti-SARS-CoV-2 S Ab, U/mL (SD)	791.2 (999.6)	119.3 (94.5)	2.90 × 10^−12^	369.8 (383.9)	261.9 (633.5)	0.0066
Anti-SARS-CoV-2 neutralizing Ab, inhibition rate, % (SD)	11.7 (4.3)	10.2 (3.8)	0.1288	15.8 (1.9)	8.8 (2.7)	2.88 × 10^−12^

Number or average values of each group are shown. Standard deviations or percentages are shown in parentheses. Differences were tested by Mann-Whitney’s U test or Fisher’s exact test using 2 × 2 contingency tables. * Fisher’s exact test was employed. Ab: antibody, S: spike, RA: rheumatoid arthritis, S high: high responders for anti-SARS-CoV-2 S Abs, S low: low responders for anti-SARS-CoV-2 S Abs, Neu high: high responders for anti-SARS-CoV-2 neutralizing Abs, Neu low: low responders for anti-SARS-CoV-2 neutralizing Abs, DMARD: disease-modifying anti-rheumatic drug, csDMARD: conventional synthetic DMARD, bDMARD: biological DMARD, and tsDMARD: targeted synthetic DMARD.

**Table 2:** *HLA* allele or haplotype carrier frequencies in RA patients with higher or lower titers of anti-SARS-CoV-2 S Abs.

	S High	S Low			
	(*n* = 22)	(*n* = 65)	*p*	OR	95% CI
DRB1*12:01	5 (22.7)	3 (4.6)	0.0225	6.08	(1.32–28.03)
DQB1*03:01	10 (45.5)	15 (23.1)	0.0583	2.78	(1.00–7.69)
DQB1*03:02	0 (0.0)	16 (24.6)	0.0089	0.07	(0.00–1.16)
DRB1*04:01-DQB1*03:01	3 (13.6)	4 (6.2)	0.3625	2.41	(0.49–11.73)
DRB1*11:01-DQB1*03:01	0 (0.0)	2 (3.1)	1.0000	0.56	(0.03–12.21)
DRB1*12:01-DQB1*03:01	5 (22.7)	3 (4.6)	0.0225	6.08	(1.32–28.03)
DRB1*12:02-DQB1*03:01	2 (9.1)	2 (3.1)	0.2641	3.15	(0.42–23.83)
DRB1*14:03-DQB1*03:01	0 (0.0)	3 (4.6)	0.5683	0.40	(0.02–7.99)
DRB1*14:06-DQB1*03:01	1 (4.5)	1 (1.5)	0.4440	3.05	(0.18–50.89)
DRB1*15:01-DQB1*03:01	0 (0.0)	1 (1.5)	1.0000	0.96	(0.04–24.31)

Top 25th percentile of the S Ab distribution was compared with the bottom 75th. Allele or haplotype carrier frequencies are shown in parenthesis (%). Associations were tested by Fisher’s exact test using 2 × 2 contingency tables. Ab: antibody, S: spike, RA: rheumatoid arthritis, OR: odds ratio, CI: confidence interval, S high: high responders for anti-SARS-CoV-2 S Abs, and S low: low responders for anti-SARS-CoV-2 S Abs.

**Table 3:** *HLA* allele or haplotype carrier frequencies in RA patients with higher or lower titers of anti-SARS-CoV-2 neutralizing Abs.

	Neu High	Neu Low			
	(*n* = 22)	(*n* = 65)	*p*	OR	95% CI
DRB1*15:01	5 (22.7)	2 (3.1)	0.0102	9.26	(1.65–52.01)
DQB1*06:02	4 (18.2)	2 (3.1)	0.0337	7.00	(1.18–41.36)
DRB1*15:01-DQB1*03:01	1 (4.5)	0 (0.0)	0.2529	9.14	(0.36–232.79)
DRB1*15:01-DQB1*06:02	4 (18.2)	2 (3.1)	0.0337	7.00	(1.18–41.36)

Top 25th percentile of the neutralizing Ab distribution was compared with the bottom 75th. Allele or haplotype carrier frequencies are shown in parentheses (%). Associations were tested by Fisher’s exact test using 2 × 2 contingency tables. Ab: antibody, RA: rheumatoid arthritis, OR: odds ratio, CI: confidence interval, Neu high: high responders for anti-SARS-CoV-2 neutralizing Abs, and Neu low: low responders for anti-SARS-CoV-2 neutralizing Abs.

**Table 4:** DRB1*12:01 and DQB1*03:01 carrier frequencies in RA patients with or without DRB1*12:01 or DQB1*03:01: associations with anti-SARS-CoV-2 S Abs.

Anti-SARS-CoV-2 S Abs	DQB1*03:01 (+)	DQB1*03:01 (−)	*p*	OR	(95% CI)
S high without DRB1*12:01	5 (29.4)	12 (70.6)	0.5052	1.74	(0.51–5.87)
S low without DRB1*12:01	12 (19.4)	50 (80.6)			
S high with DRB1*12:01	5 (100.0)	0 (0.0)	NA	NA	NA
S low with DRB1*12:01	3 (100.0)	0 (0.0)			
anti-SARS-CoV-2 S Abs	DRB1*12:01(+)	DRB1*12:01(−)	*p*	OR	(95% CI)
S high without DQB1*03:01	0 (0.0)	12 (100.0)	NA	NA	NA
S low without DQB1*03:01	0 (0.0)	50 (100.0)			
S high with DQB1*03:01	5 (50.0)	5 (50.0)	0.1936	4.00	(0.68–23.51)
S low with DQB1*03:01	3 (20.0)	12 (80.0)			

Allele carrier frequencies are shown in parentheses (%). Associations were tested by Fisher’s exact test using 2 × 2 contingency tables. RA: rheumatoid arthritis, OR: odds ratio, CI: confidence interval, NA: not applicable, S high: high responders for anti-SARS-CoV-2 S Abs, and S low: low responders for anti-SARS-CoV-2 S Abs.

**Table 5:** DRB1*15:01 and DQB1*06:02 carrier frequency in RA patients with or without DRB1*15:01 or DQB1*06:02: associations with anti-SARS-CoV-2 neutralizing Abs.

Anti-SARS-CoV-2 Neutralizing Abs	DQB1*06:02 (+)	DQB1*06:02 (−)	*p*	OR	(95% CI)
Neu high without DRB1*15:01	0 (0.0)	17 (100.0)	NA	NA	NA
Neu low without DRB1*15:01	0 (0.0)	63 (100.0)			
Neu high with DRB1*15:01	4 (80.0)	1 (20.0)	1.0000	0.60	(0.02–20.98)
Neu low with DRB1*15:01	2 (100.0)	0 (0.0)			
anti-SARS-CoV-2 neutralizing Abs	DRB1*15:01(+)	DRB1*15:01(−)	*p*	OR	(95% CI)
Neu high without DQB1*06:02	1 (5.6)	17 (94.4)	0.2222	10.89	(0.42–279.10)
Neu low without DQB1*06:02	0 (0.0)	63 (100.0)			
Neu high with DQB1*06:02	4 (100.0)	0 (0.0)	NA	NA	NA
Neu low with DQB1*06:02	2 (100.0)	0 (0.0)			

Allele carrier frequencies are shown in parentheses (%). Associations were tested by Fisher’s exact test using 2 × 2 contingency tables. RA: rheumatoid arthritis, OR: odds ratio, CI: confidence interval, NA: not applicable, Neu high: high responders for anti-SARS-CoV-2 neutralizing Abs, Neu low: low responders for anti-SARS-CoV-2 neutralizing Abs.

**Table 6:** Multiple logistic regression analysis of DRB1*12:01 and clinical manifestations: impact on anti-SARS-CoV-2 S Abs.

Anti-SARS-CoV-2 S Abs	Unconditioned			Conditioned on the Other Clinical Manifestations		
Clinical Manifestations	OR	95% CI	*p*	OR_adjusted_	95% CI	*P*_adjusted_
Age, years	0.93	(0.88–0.98)	0.0097	0.94	(0.88–0.99)	0.0328
Male	0.63	(0.19–2.12)	0.4527	0.81	(0.22–3.02)	0.7533
Corticosteroid administration	0.91	(0.34–2.49)	0.8609	0.80	(0.23–2.72)	0.7178
csDMARD administration	2.27	(0.74–6.90)	0.1499	5.18	(0.74–36.15)	0.0968
bDMARD administration	0.26	(0.03–2.17)	0.2147	0.61	(0.06–5.99)	0.6687
tsDMARD administration	0.83	(0.27–2.60)	0.7495	3.29	(0.41–26.23)	0.2611
The interval between last vaccination and blood collection, days	1.00	(0.99–1.02)	0.7881	1.00	(0.98–1.02)	0.8222
DRB1*12:01	6.08	(1.32–28.03)	0.0207	8.40	(1.29–54.77)	0.0261

Association was tested by logistic regression analysis. *p*, OR, 95% CI, *P*_adjusted_, OR_adjusted_ were calculated by logistic regression analysis on RA patients. OR: odds ratio, CI: confidence interval, DMARD: disease-modifying anti-rheumatic drug, csDMARD: conventional synthetic DMARD, bDMARD: biological DMARD, and tsDMARD: targeted synthetic DMARD.

**Table 7:** Multiple logistic regression analysis of DRB1*15:01 and clinical manifestations: impact on anti-SARS-CoV-2 neutralizing Abs.

Anti-SARS-CoV-2 Neutralizing Abs.	Unconditioned			Conditioned on the Other Clinical Manifestations		
Clinical Manifestations	OR	95% CI	*p*	OR_adjusted_	95% CI	*P*_adjusted_
Age, years	1.01	(0.96–1.07)	0.6265	1.04	(0.97–1.11)	0.2777
Male	1.70	(0.58–4.98)	0.3331	1.42	(0.40–5.09)	0.5871
Corticosteroid administration	0.70	(0.25–1.95)	0.4953	0.57	(0.17–1.98)	0.3781
csDMARD administration	1.25	(0.45–3.51)	0.6660	0.70	(0.12–4.05)	0.6891
bDMARD administration	0.26	(0.03–2.17)	0.2147	0.23	(0.02–2.31)	0.2107
tsDMARD administration	0.58	(0.17–1.95)	0.3787	0.39	(0.05–3.14)	0.3761
The interval between the last vaccination and blood collection, days	0.97	(0.95–1.00)	0.0160	0.97	(0.94–0.99)	0.0191
DRB1*15:01	9.26	(1.65–52.00)	0.0114	12.10	(1.84–79.70)	0.0095

Associations were tested by logistic regression analysis. *p*, OR, 95% CI, *P*_adjusted_, OR_adjusted_ were calculated by logistic regression analysis on RA patients. OR: odds ratio, CI: confidence interval, DMARD: disease-modifying anti-rheumatic drug, csDMARD: conventional synthetic DMARD, bDMARD: biological DMARD, and tsDMARD: targeted synthetic DMARD.

## Data Availability

Data supporting the findings of this study are presented in the paper and the Supplementary Materials. Other data are available from the authors upon reasonable request. However, the clinical information and genotype data of each participant are not available under the conditions of informed consent mandated by the Act on the Protection of Personal Information.

## Supplementary Materials

The following supporting information can be downloaded at https://www.mdpi.com/article/10.3390/vaccines11020404/s1, Table S1: DRB1 and DQB1 allele carrier frequency in RA patients with higher or lower titers of anti-SARS-CoV-2 S Abs; Table S2: DRB1 and DQB1 allele carrier frequency in RA patients with higher or lower titers of anti-SARS-CoV-2 neutralizing Abs.
