# Supplementary material for: Associations of HLA Polymorphisms with Anti-SARS-CoV-2 Spike and Neutralizing Antibody Titers in Japanese Rheumatoid Arthritis Patients Vaccinated with BNT162b2
Source: Vaccines (Basel). 2023 Feb 9;11(2):404. doi: 10.3390/vaccines11020404 (PMC9965868; doi:10.3390/vaccines11020404)
Supplement: Supplementary file 1 [file vaccines-11-00404-s001.zip › Anti-SARS-CoV-2AbRA#3-S1.pdf]

Supplementary Table S1. *DRB1* and *DQB1* allele carrier frequency in RA patients with higher or lower titers of anti-SARS-CoV-2 S Abs.

	S high (n=22)	S low (n=65)	*P*	OR	95%CI
*DRB1*01:01*	2 (9.1)	4 (6.2)	0.6402	1.53	(0.26-8.96)
*DRB1*04:01*	3 (13.6)	4 (6.2)	0.3625	2.41	(0.49-11.73)
*DRB1*04:03*	0 (0.0)	6 (9.2)	0.3304	0.20	(0.01-3.76)
*DRB1*04:05*	9 (40.9)	28 (43.1)	1.0000	0.91	(0.34-2.44)
*DRB1*04:10*	2 (9.1)	5 (7.7)	1.0000	1.20	(0.22-6.68)
*DRB1*08:03*	3 (13.6)	6 (9.2)	0.6865	1.55	(0.35-6.82)
*DRB1*09:01*	3 (13.6)	16 (24.6)	0.3776	0.48	(0.13-1.85)
*DRB1*12:01*	5 (22.7)	3 (4.6)	0.0225	6.08	(1.32-28.03)
*DRB1*13:02*	2 (9.1)	4 (6.2)	0.6402	1.53	(0.26-8.96)
*DRB1*15:01*	2 (9.1)	5 (7.7)	1.0000	1.20	(0.22-6.68)
*DRB1*15:02*	4 (18.2)	12 (18.5)	1.0000	0.98	(0.28-3.43)
*DQB1*03:01*	10 (45.5)	15 (23.1)	0.0583	2.78	(1.00-7.69)
*DQB1*03:02*	0 (0.0)	16 (24.6)	0.0089	0.07	(0.00-1.16)
*DQB1*03:03*	3 (13.6)	16 (24.6)	0.3776	0.48	(0.13-1.85)
*DQB1*04:01*	9 (40.9)	27 (41.5)	1.0000	0.97	(0.36-2.60)
*DQB1*04:02*	2 (9.1)	6 (9.2)	1.0000	0.98	(0.18-5.27)
*DQB1*05:01*	4 (18.2)	6 (9.2)	0.2648	2.19	(0.55-8.61)
*DQB1*06:01*	6 (27.3)	18 (27.7)	1.0000	0.98	(0.33-2.90)
*DQB1*06:02*	2 (9.1)	4 (6.2)	0.6402	1.53	(0.26-8.96)
*DQB1*06:04*	2 (9.1)	4 (6.2)	0.6402	1.53	(0.26-8.96)

Allele carrier frequencies are shown in parentheses (%). Association was tested by Fisher's exact test using 2X2 contingency tables. Alleles with more than 5% of the carrier frequency in RA are shown. Ab: antibody, S: spike protein, RA: rheumatoid arthritis, OR: odds ratio, CI: confidence interval, S high: high responders for anti-SARS-CoV-2 S Abs, S low: low responders for anti-SARS-CoV-2 S Abs.
