# Supplementary material for: Associations of HLA Polymorphisms with Anti-SARS-CoV-2 Spike and Neutralizing Antibody Titers in Japanese Rheumatoid Arthritis Patients Vaccinated with BNT162b2
Source: Vaccines (Basel). 2023 Feb 9;11(2):404. doi: 10.3390/vaccines11020404 (PMC9965868; doi:10.3390/vaccines11020404)
Supplement: Supplementary file 1 [file vaccines-11-00404-s001.zip › Anti-SARS-CoV-2AbRA#3-S2.pdf]

Supplementary Table S2. *DRB1* and *DQB1* allele carrier frequency in RA patients with higher or lower titers of anti-SARS-CoV-2 neutralizing Abs.

	Neu high (n=22)	Neu low (n=65)	*P*	OR	95%CI
*DRB1*01:01*	2 (9.1)	4 (6.2)	0.6402	1.53	(0.26-8.96)
*DRB1*04:01*	1 (4.5)	6 (9.2)	0.6735	0.47	(0.05-4.12)
*DRB1*04:03*	1 (4.5)	5 (7.7)	1.0000	0.57	(0.06-5.18)
*DRB1*04:05*	10 (45.5)	27 (41.5)	0.8058	1.17	(0.44-3.10)
*DRB1*04:10*	1 (4.5)	6 (9.2)	0.6735	0.47	(0.05-4.12)
*DRB1*08:03*	0 (0.0)	9 (13.8)	0.1042	0.13	(0.01-2.37)
*DRB1*09:01*	3 (13.6)	16 (24.6)	0.3776	0.48	(0.13-1.85)
*DRB1*12:01*	3 (13.6)	5 (7.7)	0.4114	1.89	(0.41-8.68)
*DRB1*13:02*	3 (13.6)	3 (4.6)	0.1669	3.26	(0.61-17.52)
*DRB1*15:01*	5 (22.7)	2 (3.1)	0.0102	9.26	(1.65-52.01)
*DRB1*15:02*	3 (13.6)	13 (20.0)	0.7513	0.63	(0.16-2.46)
*DQB1*03:01*	8 (36.4)	17 (26.2)	0.4175	1.61	(0.58-4.52)
*DQB1*03:02*	3 (13.6)	13 (20.0)	0.7513	0.63	(0.16-2.46)
*DQB1*03:03*	3 (13.6)	16 (24.6)	0.3776	0.48	(0.13-1.85)
*DQB1*04:01*	10 (45.5)	26 (40.0)	0.8028	1.25	(0.47-3.31)
*DQB1*04:02*	1 (4.5)	7 (10.8)	0.6735	0.39	(0.05-3.40)
*DQB1*05:01*	4 (18.2)	6 (9.2)	0.2648	2.19	(0.55-8.61)
*DQB1*06:01*	3 (13.6)	21 (32.3)	0.1057	0.33	(0.09-1.24)
*DQB1*06:02*	4 (18.2)	2 (3.1)	0.0337	7.00	(1.18-41.36)
*DQB1*06:04*	3 (13.6)	3 (4.6)	0.1669	3.26	(0.61-17.52)

Allele carrier frequencies are shown in parentheses (%). Association was tested by Fisher's exact test using 2X2 contingency tables. Alleles with more than 5% of the carrier frequency in RA are shown. Ab: antibody, RA: rheumatoid arthritis, OR: odds ratio, CI: confidence interval, Neu high: high responders for anti-SARS-CoV-2 neutralizing Abs, Neu low: low responders for anti-SARS-CoV-2 neutralizing Abs.
